# Development and Implementation of the “Exercise is Medicine” Elective at an Osteopathic Medical School

**DOI:** 10.1007/s40670-024-02172-z

**Published:** 2024-10-25

**Authors:** Cameron Mitchell, Samantha DeMartino, Lauren Ivers, Abigail Brown, Emily Maccabee, Brian Griffith, Christopher L. Pankey

**Affiliations:** Department of Biomedical Sciences, West Virginia School of Osteopathic Medicine, 400 Lee St, Lewisburg, WV 24901 USA

**Keywords:** Medical education, Lifestyle modifications, Physical activity, Curriculum development, Disease prevention, Exercise

## Abstract

**Introduction:**

Lifestyle changes are a powerful way to combat many chronic illnesses. However, education on appropriate instruction of physical activity appears to be insufficient among medical students. This manuscript highlights development, incorporation, and feedback of the Exercise is Medicine elective at the West Virginia School of Osteopathic Medicine.

**Materials and Methods:**

Twenty-four medical students attended ten weekly sessions, each of which lasted 2 h and covered a different topic. Participants were selected based on previous experience with exercise-based medicine and their personal beliefs on incorporating it into clinical encounters. Outcomes were gathered using surveys consisting of the Likert Scale, rating, yes/no/maybe, and free text questions.

**Results:**

Confidence in prescribing lifestyle changes increased (*p* < 0.0001) from pre- to post-elective. Feelings of incorporating course material into practice increased (*p* < 0.05) after the elective. Feelings that physicians could impact their patients’ physical activity did not change.

**Conclusion:**

Implementation of the Exercise is Medicine elective was well received among students and increased student knowledge and confidence in prescribing lifestyle changes to a variety of patient populations.

**Supplementary Information:**

The online version contains supplementary material available at 10.1007/s40670-024-02172-z.

## Introduction

In the United States, 60% of adults are estimated to suffer from a chronic disease, while 40% suffer from two or more [1]. These alarming rates are the primary contributors to the nation’s 4.1 trillion dollars ($12,000 per person) of annual health care costs, and encompass a variety of conditions including metabolic, cardiovascular, neurologic, psychological, and musculoskeletal diseases [1]. Although these diseases affect a broad scope of physiologic systems, they share causal factors, most of which are consequences of lifestyle. Therefore, lifestyle modifications can contribute to disease prevention, provide lasting therapeutic effects, and even be curative for some chronic diseases.

Arguably, one of the most powerful lifestyle changes we can implement to prevent and treat chronic disease is increased physical activity (PA) or exercise [2]. Increasing PA is the most common modifiable risk factor for preventable chronic diseases and has been shown to directly prevent 8 out of 10 leading causes of mortality in the US [3, 4]. Over the past few decades, many scientific societies and organizations have promoted physical activity and exercise recommendations for policy change or advocated for the clinical prescription of PA for disease prevention and treatment [5–9]. Because of these efforts, exercise prescription is considered a first-line therapy in the prevention and treatment for 26 chronic diseases [10]. However, only 23% of Americans meet current exercise recommendations [11], and 12.5% of physician office visits include physical activity counseling [12]. Together, these studies suggest that despite the therapeutic effectiveness of physical activity, there are considerable barriers to implementing it into clinical practice. Physicians have noted a lack of significant training in lifestyle interventions [12], which contributes to decreased confidence in providing safe, effective exercise recommendations to their patients.

In general, the current treatments for chronic diseases include pharmaceuticals, diet and exercise recommendations, behavioral modification, and surgical intervention [13]. Despite these efforts, disease rates have increased over the past few decades and this elevation is projected to continue [14]. The rising disease rates coupled with deficient PA counseling in clinical practice have presented the opportunity to find ways to advance existing therapeutic measures. The authors feel that one way to combat this is to provide adequate understanding of the therapeutic effectiveness of physical activity.

In response, the West Virginia School of Osteopathic Medicine has established recognition from the American College of Sports Medicine as a 2023 Gold Campus for its efforts in promoting Exercise is Medicine—On Campus®. Part of this effort included creating educational opportunities for students, such as an elective course, to expand their knowledge of the benefits of being physically active. As future practitioners of evidence-based medicine, it is important that medical students learn the evidence behind the efficacy of physical activity. Medical students must also gain the knowledge necessary to avoid the potential dangers of certain exercise modalities and practices in various disease states. In other words, although exercise and PA are generally accepted as beneficial practices, patients with chronic disease require specific considerations to both maximize the efficacy of PA and simultaneously avoid disease complications, injury, or other adverse outcomes.

This manuscript outlines the development of the Exercise is Medicine (EIM) elective at the West Virginia School of Osteopathic Medicine and provides feedback from the inaugural class of medical students. The authors hypothesized that the elective would be in high demand, would add to the student’s perceived breadth of medical education, and would be accepted as beneficial for medical training.

## Materials and Methods

### Curriculum Development

The course curriculum was developed by three medical school faculty members and five medical students who had backgrounds in exercise physiology. The curriculum was developed using American College of Sports Medicine (ACSM) Clinical Exercise Physiology and ACSM Exercise Testing and Prescription textbooks and other current evidence-based guidelines [15, 16]. The course consisted of 2-h-long sessions occurring weekly over 10 weeks. The first four sessions covered the foundations of EIM, while the last six sessions focused on applying EIM to specific patient populations (Table 1). Each session began with an hour of lecture-style didactics, followed by an hour of applied learning in the form of demonstrations, small-group discussions, and interactive sessions. At the end of the course, students applied their knowledge and demonstrated their competency in a final capstone project.

**Table 1:** Outline of the topics included in the EIM elective

Foundations of Exercise is Medicine	
Exercise is Medicine introduction	Goals and background of EIM, EIM-on campus, and exercise prescription
Exercise basics and exercise testing	Estimating and evaluating cardiorespiratory fitness from exercise and body composition tests. Using various exercise modalities to personalize exercise in accordance with safe exercise guidelines
Behavior modification in patient encounters	Discussing transtheoretical model and strategies to overcome barriers to exercise with patients
Incorporating EIM into a clinical setting	Including physical activity as vital sign (PAVS), benefits, and risks of exercise, fitting EIM into various specialties
Exercise prescription of specific populations	
Diabetes and metabolic syndrome	Exercise modalities and their effects on blood glucose, obesity, weight loss, and metabolic syndrome
Cardiovascular disease	Deter public misconceptions on physical activity exacerbations and promote physiologic changes to prevent disease progression
Neuromuscular disease	Modifications to exercise and strength training to combat deficits after stroke Benefits of multimodal exercise, specifically boxing, in Parkinson’s disease, dementia, and Alzheimer’s disease
Pulmonary disorders	Exercise induced improvement in respiratory health and improvements in oxygen utilization
Immunocompromised states	Changes in leukocytes with exercise. Increased benefits in cancer therapy and prognosis including pre-habilitation
Children and elderly	Targeted exercises for both populations including increased bone mass, balance, and cognition

The learning outcomes of the course were for the students to be able to:

1. Demonstrate appropriate use of exercise guidelines and tests as foundations to medical care, disease prevention, and health promotion.
2. Describe motivational interview skills that physicians utilize to engage with patients and family for positive behavior changes.
3. Identify and practice history taking and physical examination skills that relate specifically to lifestyle-related health status. These include vital signs, diet, physical activity, stress levels sleep patterns, and sense of well-being.
4. Construct an evidence-based, achievable, specific, written action plan and develop exercise prescriptions.
5. Identify appropriate use of community resources that support the implementation of healthy lifestyles.

The capstone project was a timed standardized patient encounter in which students were asked to gather a history and develop an assessment and plan for the patient using exercise prescriptions and other principles of EIM. The students worked in teams of two. First-year students were paired with second-year students when possible. The assignment began with a 15-min “briefing” to provide context for the upcoming clinical scenario. The encounter mimicked a follow-up appointment at a general outpatient clinic, where the patient was seeking lifestyle advice following a diagnosis of multiple comorbid chronic conditions. Broadly, patients presented with a combination of two or more of the following: obesity/overweight, hypertension, hypotension, heart disease, osteoporosis, arthritis, depression, or anxiety. Students were given time to review pertinent patient information such as vital signs and laboratory results and develop a plan for their patient encounter. Following the orientation, the students engaged in a 15-min patient encounter during which they were expected to demonstrate the same clinical skills required for a graded activity in their medical curriculum. They were also instructed to ensure that the patient knew and understood the plan devised during the encounter. The standardized patients (SP) were trained to know their medical history and be open to or request information about physical activity. Some SPs were vague in their requests, while others asked more detailed questions such as “Can I perform heavy weightlifting?”, or similar queries. After the encounter, students were given 10 min to document the visit in a standard SOAP note. The capstone project concluded with 30-min debriefing sessions [17]. The debriefing session included a summary of the patient presentations and a discussion regarding the indicated physical activity and important contraindications for each scenario and chronic disease course. Additional discussion focused on implementation of motivational interviewing and strategies for discussing sensitive topics such as body weight with patients.

The curriculum was approved by the WVSOM Curriculum Committee, and students were awarded 1.5 credit hours at completion of the course. The elective was structured as pass/fail. Students needed to participate in class discussions, record attendance, and complete the SP encounter to earn a mark of “pass” which was then reported to the Registrar’s Office for transcript reporting.

### Participants

The course was limited to 25 first- and second-year medical students during the 2022–2023 academic year. Students who expressed interest in the elective were asked to complete an application that consisted of providing information about any previous experience with exercise medicine and their thoughts on the importance of integrating it into clinical encounters. Students signed up using a circulated sign-up link that was sent via email to all first- and second-year students on campus.

Students who participated in the elective were asked to complete a pre-course and post-course evaluation (Supplementary Information Table 1). The surveys utilized the Likert Scale, ratings, yes/no/maybe, and free text questions. The 6-question pre-course survey evaluated baseline knowledge of EIM, understanding on the use of EIM in clinical practice, and current personal physical activity habits. The 19-question post-course survey included all questions from the first survey, plus additional questions that assessed the students’ perceived level of knowledge acquired from the elective, and the student’s perceived confidence in applying EIM to future clinical scenarios. The post-survey also allowed students to share their honest feedback and provide suggestions for future versions of the EIM elective course.

Study data were collected and managed using REDCap electronic data capture tools hosted at WVSOM [18, 19]. REDCap (Research Electronic Data Capture) is a secure, web-based software platform designed to support data capture for research studies, providing (1) an intuitive interface for validated data capture; (2) audit trails for tracking data manipulation and export procedures; (3) automated export procedures for seamless data downloads to common statistical packages; and (4) procedures for data integration and interoperability with external sources. This study was reviewed by the WVSOM Institutional Review Board (protocol number 2022–13) and declared Exempt.

### Data and Statistical Analysis

**Fig. 1:**
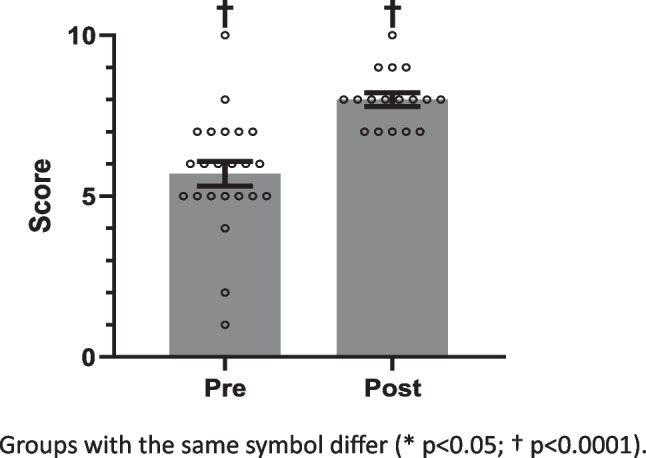
Pre- and post-elective levels for perceived confidence regarding lifestyle prescriptions for chronic disease

**Fig. 2:**
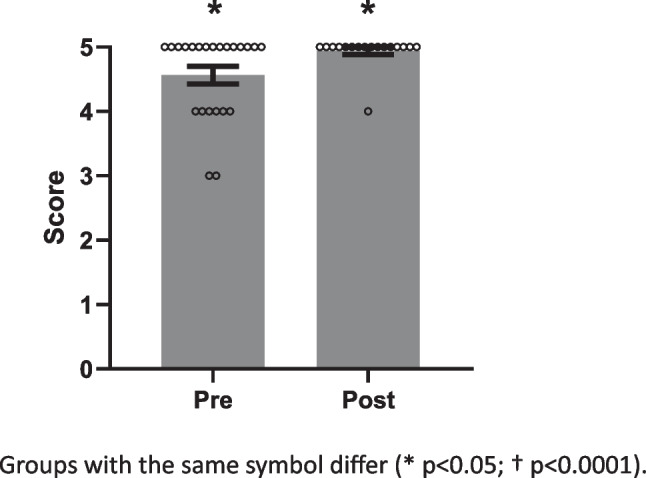
Feeling that course material should be incorporated into practice

**Fig. 3:**
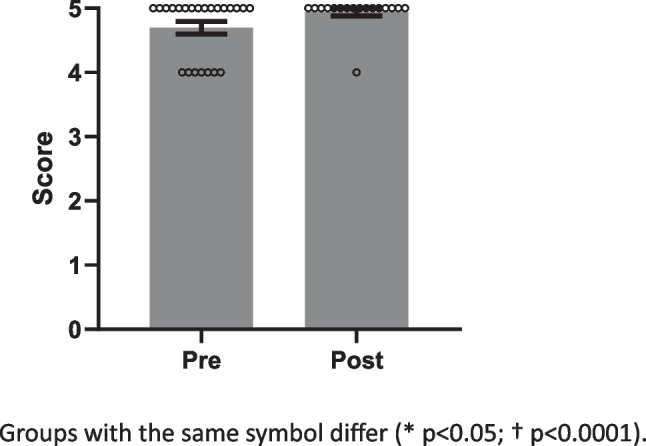
Feeling that physicians could impact their patients’ physical activity

Likert scales from 1 to 10 were used to assess confidence in lifestyle medicine, relevance of course content to physician roles, and how likely student doctors were to implement the material into their future practice (Fig. 1), with 1 indicating a low score and 10 indicating a high score. Assessment of agreements with statements (Figs. 2 and 3) about inclusion of course content into clinical practice and physicians’ effect on patients’ physical activity were collected as Strongly Disagree, Disagree, Neutral, Agree, or Strongly Agree. These answers were transformed to ordinal data from 1 to 5, with Strongly Disagree corresponding to 1, ascending to Strongly Agree = 5. The data from the pre- and post-surveys were compared using one-tailed Mann–Whitney tests. Questions asked only after the elective was completed (Fig. 3) were collected as Likert data similar to the above and summarized as mean ± SEM. Alpha was set at 0.05 for all statistical comparisons.

## Results

### Enrollment

Twenty-four students expressed interest and enrolled in the course. Nineteen were first-year, and five were second-year osteopathic medical students. No student failed this elective.

### Pre- and Post-Course Survey

Twenty-three students completed the pre-course survey, and 17 students completed the post-course survey. Confidence in prescribing lifestyle changes increased (*p* < 0.0001) from pre- to post-elective (Fig. 1). Similarly, feeling that course material should be incorporated into practice increased (*p* < 0.05) after the elective (Fig. 2), but believing that physicians could impact their patients’ physical activity did not change (Fig. 3). Finally, post-course surveys revealed perceived relevance of the elective material had a mean of 9, and a median of 10. Similarly, the likelihood to incorporate the material into future practice had a mean of 9.5, and a median of 10. One hundred percent of the students agreed that they would recommend this elective for their colleagues.

### Standardized Patient Encounter Capstone Project

Sixteen (of 17) respondents of the post-course survey rated the SP encounter a 10/10, and one student rated it an 8/10. The SP encounter was the highest rated part of the elective and was given additional praise in the open comment section by over 1/3 of respondents.

## Discussion

The EIM elective at the West Virginia School of Osteopathic Medicine (WVSOM) successfully enhanced first- and second-year medical students’ knowledge and confidence in prescribing physical activity. Data from the pre- and post-course surveys revealed a significant increase in student’s perceived relevance of course material and ability to prescribe exercise as a therapeutic option. Free response feedback (see supplementary information) further highlighted these perceived benefits and offered insights into areas for improvement.

The increase in confidence observed from the survey data suggests that the elective effectively met its objective of bolstering students’ capabilities in prescribing exercise. This aligns with previous research indicating that targeted educational interventions can improve medical students’ competence in lifestyle medicine [20]. The high ratings for the standardized patient encounter underscore its value in bridging theoretical knowledge with practical application, a finding consistent with studies emphasizing the importance of experiential learning in medical education [21].

Qualitative feedback revealed several key themes. First, students felt more prepared to integrate exercise prescriptions in patient care, reflecting the course’s success in addressing this gap in medical education. The capstone project was particularly enjoyed for its real-world applicability, reinforcing the need for practical, case-based learning experiences.

The content was delivered via traditional lecture style incorporating group discussions and activities to reinforce the material. Based on feedback collected from the surveys, this style of teaching was praised by the students. Overall, the survey data suggest that the elective was well received and beneficial for medical training. Students who completed the survey recommend this elective to their colleagues, indicating a potential demand for similar programs in medical education. This was reflected in the three-fold increase in the number of participants that registered for the elective in the next academic year.

The SP encounter was highly praised by the participating students in the course survey and was described as an enjoyable and informative learning experience. The authors intentionally paired second-year students with first-year students to ensure a uniform skill and experience level across all groups. Further, this encounter was not a graded activity as it was not feasible to hold varying experience levels to the same standard. Despite this, students were still encouraged to perform best-practices. To avoid the development of unfavorable habits, the importance of performing to the standards required by the main clinical skills curriculum was emphasized to the students during the briefing. In hindsight, these were good decisions which facilitated an equitable learning environment.

During the elective, disease states were typically presented individually. However, in the SP encounter, students were faced with the challenge of multi-morbidities and had to create an activity plan that was safe and effective within the confines of the patients’ abilities. This created a challenging learning environment, forcing students to implement the highest level of Bloom’s taxonomy and create a treatment plan based on the knowledge they had acquired over the span of the elective [22].

There were limitations to this study. The small sample size confined to WVSOM students limits the generalizability of the findings. Not all students completed the pre- and post-elective surveys as instructed, decreasing numbers further. Additionally, the short-term nature of the study limits the representation of long-term influence on students’ clinical practices. Another perceived limitation of the course was the lecture-style delivery of information each week. Based on student feedback and our team’s consensus, the course had extraneous information that detracted from the elective outcomes. Specifically, much of the content delivered in the lectures was undergraduate or master’s level exercise physiology. While this information is fundamental to the students understanding, many of the students had previously taken exercise physiology courses in their education prior to matriculation at WVSOM. Therefore, the authors suspect that more focus on the medical-level application of exercise physiology will be of greater benefit to future students. Moving forward, the authors intend to reduce the time spent on background information and increase the focus on patient presentations, and the indicated and contraindicated activities unique to diseased populations.

In the course’s future iterations, the curriculum will incorporate case studies with application exercises emphasizing the information. The course will be streamlined to maintain the integrity of information being delivered. The future iterations of the course intend to improve upon the previous by maintaining student engagement for the entire duration of the course.

## Conclusions

This manuscript outlines the development and implementation of the Exercise is Medicine elective, providing valuable insight into the potential benefits of incorporating lifestyle medicine into medical education. The positive student feedback and increased confidence in prescribing lifestyle changes suggest that such initiatives can contribute to addressing the gap in physicians’ training related to lifestyle therapies. Future studies with larger sample sizes and extended follow-up periods could further validate the efficacy and sustainability of integrating such electives into medical curricula. Overall, this manuscript is a valuable contribution to the ongoing dialogue on enhancing medical education to better address the growing burden of chronic diseases through lifestyle interventions.

## Supplementary Information

Below is the link to the electronic supplementary material.

Supplementary file1 (DOCX 33 KB)
