# Supplementary material for: Development and Implementation of the “Exercise is Medicine” Elective at an Osteopathic Medical School
Source: Med Sci Educ. 2024 Oct 25;35(1):227–32. doi: 10.1007/s40670-024-02172-z (PMC11933613; doi:10.1007/s40670-024-02172-z)
Supplement: Supplementary file 1 — Supplementary file1 (DOCX 33 KB) [file 40670_2024_2172_MOESM1_ESM.docx]

Supplementary Information for: *Development and Implementation of the ‘Exercise is Medicine’ Elective at an Osteopathic Medical School*

Pre- and Post-Course Survey Questions

| **Pre-Course Survey** |
| --- |
| 1. Exercise is medicine should be included in any routine appointment just like diagnosis and treatment.    1. *Strongly agree, Agree, Neutral, Disagree, Strongly Agree* 2. Physicians can have an effect on a patient’s physical activity if they take the time to discuss the problem.    1. *Strongly agree, Agree, Neutral, Disagree, Strongly Agree* 3. Physicians should include exercise is medicine in their clinical practice.    1. *Strongly agree, Agree, Neutral, Disagree, Strongly Agree* 4. Prior to taking the exercise is medicine elective, how would you rate your confidence level to lifestyle treatment options.    1. *Scale 0-10* 5. On average, how many days per week do you engage in moderate to vigorous physical activity (like a brisk walk)?    1. *1 day – 7 days* 6. On average, how many minutes do you engage in physical activity at the level?    1. *Free text* |
| **Post-Course Survey** |
| 1. After completing this course, how would you rate your confidence level regarding exercise is medicine?    1. *Scale 0-10* 2. Exercise is medicine should be included in any routine appointment.    1. *Strongly agree, Agree, Neutral, Disagree, Strongly Agree* 3. Physicians should include exercise is medicine in their clinical practice.    1. *Strongly agree, Agree, Neutral, Disagree, Strongly Agree* 4. Physicians can have an effect on a patient’s physical activity if they take the time to discuss the problem.    1. *Strongly agree, Agree, Neutral, Disagree, Strongly Agree* 5. How relevant is this course to your role as a physician?    1. *Scale 0-10* 6. How clear were the course objectives?    1. *Scale 0-10* 7. How would you rate the lecture content in this course?    1. *Scale 0-10* 8. How would you rate the application exercises?    1. *Scale 0-10* 9. How would you rate the hands-on exercise activities in this course?    1. *Scale 0-10* 10. How would you rate the final standardized patient encounter in this course?     1. *Scale 0-10* 11. How likely are you to incorporate what you learned into your clinical practice?     1. *Scale 0-10* 12. How do you feel about the amount of information presented in this course?     1. *Scale 0-10* 13. Compared to the other courses you have taken in your career, how would you rate: this course increased my knowledge of exercise     1. *Strongly agree, Agree, Neutral, Disagree, Strongly Agree* 14. Compared to the other courses you have taken in your career, how would you rate: this course increased my knowledge of exercise treatment options for patients     1. *Strongly agree, Agree, Neutral, Disagree, Strongly Agree* 15. What did you like most about this course?     1. *Free text* 16. What would you change in this course?     1. *Free text* 17. Would you recommend this Exercise is Medicine elective to a colleague?     1. *Yes, no, maybe* 18. On average, how many days per week do you engage in moderate to vigorous physical activity (like a brisk walk)?     1. *1 day – 7 days* 19. On average, how many minutes do you engage in physical activity at the level?     1. *Free text* |

Answers to Free-text survey questions in the post-course survey

| 15. What did you like most about this course? |
| --- |
| I like that it was split into patient populations and that each course director brought a unique view and knowledge to the course - it felt like all the based were covered. |
| I really liked that we looked at different patient populations. |
| The SP encounter! It was a nice culmination for the course. |
| Patient encounter |
| I enjoyed the information on how to bring up these difficult topics with patients and specific things to avoid based on diagnosis. I have no background in EIM so this was very helpful. |
| I really liked when we were given specific do’s and don’ts for each type of patient presentation. |
| Learning how to talk to patients about beginning exercise. Knowing what to look for when starting an exercise program. |
| The practical aspect of learning how exercise can help every patient. |
| I enjoyed being introduced to the various exercise modalities and measurements (VO2max) as well as how different clinical presentations can modify the treatment plan. |
|  |
| Hearing from a variety of students and faculty that come from different professional perspectives. |
| I enjoyed the applicability to real life scenarios. |
| The SP |
| The SP encounter at the end. It was helpful to get a real understanding of what an appointment could look like and how to ask questions/get responses/come up with ideas for unique populations. It was also helpful practice to think of how different disease states can contribute to recommendations you choose to make or advise against. |
| I enjoyed the SP encounter since we got to apply what we learned in class. I also enjoyed some of the more interactive parts of the course like learning how to measure body fat and making fitness prescriptions. |
| lectures on exercise function tests. |
| The patient encounter was a great way to apply what we learned and I felt like I got the most out of the course through that interaction. |

| 16. What would you change in this course? |
| --- |
| I would have liked to have more patient SP encounters to practice incorporating EIM knowledge. Also, during class I would have liked more interactive activities/visuals- for example instead of telling me how great boxing is for Parkinson’s - maybe you could also include a video/visual to show us the benefits of a Parkinson’s patient boxing/exercising. |
| I would’ve liked more clinical applications. It was nice to understand the Phys behind why we were making these recommendations but there could have been a higher focus on how to actually transfer this information to our patient. |
| N/A |
| Lectures were very informative, but maybe a little long and verbose. |
| There were some weeks where it was difficult to attend due to outside class material and scheduling |
| I think having more time to work with what we’ve learned each week would be helpful. For example having time to create prescriptions like we did one of the earlier weeks. |
| I would add a lecture on different types of diets. There are so many "fad" diets out there that people are getting into. I would like to know what's current and the literature on those diets. for example, when we talk about Mediterranean, DASH, Keto, paleo, diets like these I would like to learn more about. This would help understanding when recommending diets and also understanding the patient better |
| Maybe talk to dieticians, physical therapists, or other health care providers. |
| The setup is great. This is more of a personal pet peeve, but I believe focusing on creating presentations with less words and more images/charts/diagrams/digested schematics helps with long term comprehension. I understand this is difficult; since presenting research findings requires a lot of writing. But if it was possible, this is what I would try to do. Overall l, I loved this course!! |
| I would like there to be more specific examples of what we can advise/counsel the patients on doing for exercises such as specific modifications for disease populations. I think some patients may not go forward with exercising if they have to add on additional appointments with another health professional. |
| Make the course shorter if possible |
| Nothing, I thought it was structured will and it was nice how we got to implement the topic we just went over the same day. |
| Length of lectures. I think it would have been better to have more but shorter lectures |
| Implementing more examples of special population patients. It would have been helpful to have real examples of "this person came in with x, this is the regimented plan that he was on, here's what we can do and here's what we might want to avoid." |
| Some of the lectures were too long and did not have an interactive component. It would be nice to have more application-related activities, more SP encounters, or making more fitness prescriptions for patients. Wish the class was during day-time hours vs in the evening. |
| More lectures from content experts. |
| Maybe have less review on each body system that was covered. |
